# Subregional analysis of joint stiffness facilitates insight into ligamentous laxity after ACL injury

**DOI:** 10.3389/fbioe.2023.1298402

**Published:** 2023-12-22

**Authors:** Danni Wu, Xuan Zhao, Bin Wu, Lan Zhou, Ye Luo, Xiaofan Huang, Weidong Xu, Shaobai Wang

**Affiliations:** ^1^ Key Laboratory of Exercise and Health Sciences of Ministry of Education, Shanghai University of Sport, Shanghai, China; ^2^ Department of Orthopedics, Changhai Hospital, The Navy Medical University, Shanghai, China

**Keywords:** anterior cruciate ligament, knee laxity, stiffness, regional analysis, load-displacement curve

## Abstract

**Purpose:** Increased incidence of anterior cruciate ligament injuries has amplified the need for quantitative research in clinical and academic settings. We used a novel digital arthrometer to measure knee laxity in healthy people and patients with anterior cruciate ligament injuries. Changes in stiffness were also assessed to develop new indicators for detecting anterior cruciate ligament injury. The purpose of this study was to use arthrometer to measure the quantitative indicator of knee laxity, bringing clinicians a new perspective on how to identify injury to the ACL.

**Methods:** In this cross-sectional study, anterior tibial displacement under continuous loading was measured using a novel digital arthrometer in 30 patients with unilateral anterior cruciate ligament injury and 30 healthy controls. Load-displacement curves were plotted, using real-time load and displacement changes. Stiffness was defined by the slope of the applied load to tibial displacement. Anterior tibial displacement and instantaneous stiffness values under different loads were compared. The restricting contribution of the anterior cruciate ligament transformed the displacement-stiffness curve from a sharp decrease to a stable increase, resulting in a minimum stiffness value. Using the minimum stiffness as the turning point, the load-displacement curve was divided into regions 1 and 2. The two regions’ stiffness changes were compared. Based on the findings, receiver operating characteristic curves were plotted and the area under the curve was calculated to estimate the diagnostic accuracy.

**Results:** Anterior tibial displacement was significantly greater in the anterior cruciate ligament injury group than in the controls under each 10-N increase load (*p* < 0.05). In the anterior cruciate ligament injury group, instantaneous stiffness was significantly lower on the injured side than on the healthy side (*p* < 0.05). In the two regions of the load-displacement curve, stiffness was significantly lower in the anterior cruciate ligament injury group than in the control group (all, *p* < 0.05). Receiver operating characteristic curves were plotted, using changes in stiffness under the two regions in both groups. Stiffness in region 2 had the largest area under the curve (0.94; 95% CI, 0.88–0.99). Using the cut-off value of 9.62 N/mm to detect ACL injury, the sensitivity and specificity were 93% and 82%, respectively.

**Conclusion:** Our investigation of ligament stiffness provides novel insights into the properties of knee laxity. Stiffness in the later stages of increased loading <9.62 N/mm could be a valid indicator for identifying knee laxity.

## Introduction

An accurate assessment of knee laxity is essential for the treatment and rehabilitation evaluation of anterior cruciate ligament (ACL) injuries. Physical examination is a convenient and rapid diagnostic tool frequently used to achieve this assessment in clinical practice (IW et al., 1989; Benjaminse et al., 2006). The Lachman test is an effective tool for examining anterior tibial translation, with studies indicating a sensitivity of 81%–94% and a specificity of 81%–98% (van Eck et al., 2013b; Rohman and Macalena, 2016). However, this test is influenced by a clinician’s experience and a patient’s muscle tone (IW et al., 1989; Benjaminse et al., 2006). Arthrometers are devices that apply a repeatable load to the knee joint and mechanically measure the resulting displacement (IW et al., 1989; Benjaminse et al., 2006). Measurement with these devices, compared to a physical examination, provides more objective, quantitative, and accurate information. An arthrometer is a commonly used clinical instrument for the objective assessment of ACL injuries (Pugh et al., 2009; Rohman and Macalena, 2016).

Several types of arthrometers exist. For example, the KT1000 (MED Metric Corp., San Diego, CA, United States) has commonly been used to evaluate ACL injuries (Küpper et al., 2007). In 1985, the KT2000 arthrometer was first used by researchers to visualize load-displacement curves to reflect the differences in relaxation with increasing load (Daniel et al., 1985b). Stiffness is defined by the slope of the applied load to the tibial displacement (Markolf et al., 1976). It is often used to reflect the biomechanical properties of ligaments (Woo et al., 1991). As the load increases, the stiffness changes, thereby representing a different condition of the ligament. However, previous studies that used the KT1000 or KT2000 have demonstrated that the application of load was typically discontinuous during the test. By contrast, (IW et al., 1989) suggested that discrepancies exist in the retest results of this device. For knee laxity measurements, the KiRA (I+, Italy) and the GNRB (Genourob, Laval, France) have recently been certified to be comparable to the KT-1000. The KiRA is a device that measures knee rotational and translational laxity using triaxial accelerometers and gyroscopes (Raggi et al., 2019). In a study of reliability, Runer et al. (2021) evaluated the measurements of four arthrometers (KT-1000, Rolimeter, KLT and Kira). The results showed that the KiRA device measured higher values than the other devices and had a 34% false positive rate. The GNRB is a computerized device that uses pressure and motion sensors to evaluate knee laxity automatically in a consistent direction and speed (Smith et al., 2022). However, Mouarbes et al. (2018) discovered poor retesting consistency while investigating the reliability of the GNRB for testing knee relaxation in healthy adults. Therefore, the problem of how to improve the accuracy of arthrometer measurements remains to be resolved.

A recent novel arthrometer (Ligs, Innomotion, Shanghai, China), has built-in load and displacement sensors to continuously record real-time load and displacement with an accuracy of 1 N and 0.1 mm. Ligs is optimized in terms of structure and measurement methodology. The interrater reliability and the intrarater reliability were considered excellent with ICC score of 0.91 and 0.94. The sensitivity and specificity of Ligs for the identification of ACL injuries were 87% and 73% at a load of 150N (Wu et al., 2023). In the present study, we compared differences in knee laxity between healthy individuals and patients with ACL injuries by using this novel digital arthrometer. We hypothesized that a significant difference would exist in knee laxity between healthy individuals and patients with ACL injuries. When arthrometer is used to measure knee laxity, a gradual increasing load is accompanied by an initial directional compression of the knee joint. Subsequently the ACL acts to initiate a restrictive effect on the tibia (Maitland et al., 1995). We divide the load-displacement curves into different regions based on the above two phases and then analyze the stiffness changes in these two regions. Therefore, we propose a novel objective indicator to identify knee laxity by analyzing stiffness changes in different regions. We hypothesized that these two regions of stiffness variation would show different characteristics between healthy individuals and patients with ACL injuries.

## Materials and methods

### Participants

Thirty patients with unilateral ACL injuries were enrolled as the study group. Participants in the ACL injury group were identified by sports medicine specialists based on clinical symptoms and magnetic resonance imaging (MRI) results. MRI images demonstrated increased T2 signal, diffuse thickening, and structural disturbances in the ACL. In addition, the Lachman test results were positive. Furthermore, 30 healthy participants were included as a control group. All healthy participants were subjected to a physical examination to ensure that their knee joints were in good functioning condition. The study was conducted in accordance with the Declaration of Helsinki and was approved by the ethics committee of our institution (approval no. 1XXXXXXXXXXX0). All participants signed an informed consent form before participation in the trial.

### Data acquisition

**FIGURE 1:**
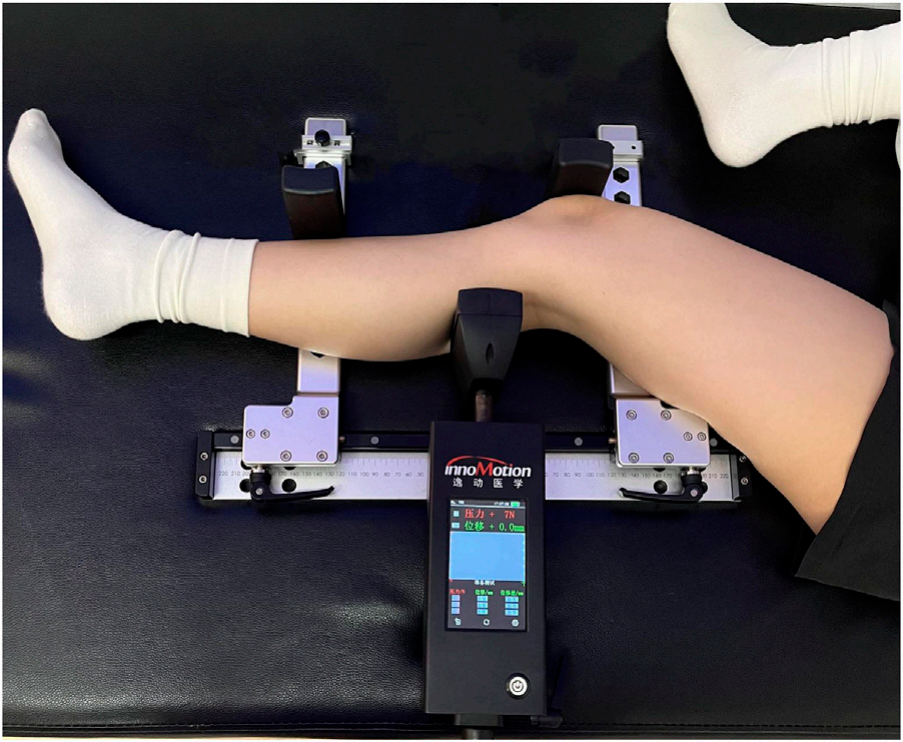
Measurement of anterior tibial displacement (ATD), using the Ligs (Innomotion, Shanghai, China). The lower leg is fixed by the patella and the distal tibial component. The tibia is displaced anteriorly by pushing on the thruster located at the posterior lower leg.

The Ligs arthrometer was used to assess knee laxity under continuous loads. Load and displacement were input into the Ligs storage unit by using digital sensor with a sampling rate of 30Hz. Displacement recording was initiated when the load exceeded 20 N to attenuate the impact of muscle tissue (Wu et al., 2023). The load is accurate to 1 N, and the displacement is accurate to 0.1 mm. Patients were required to maintain continuous muscle relaxation throughout the duration of the test. After fixing the lower leg, the tibia was displaced anteriorly by pushing the thruster that was located behind the lower leg (Figure 1).

**FIGURE 2:**
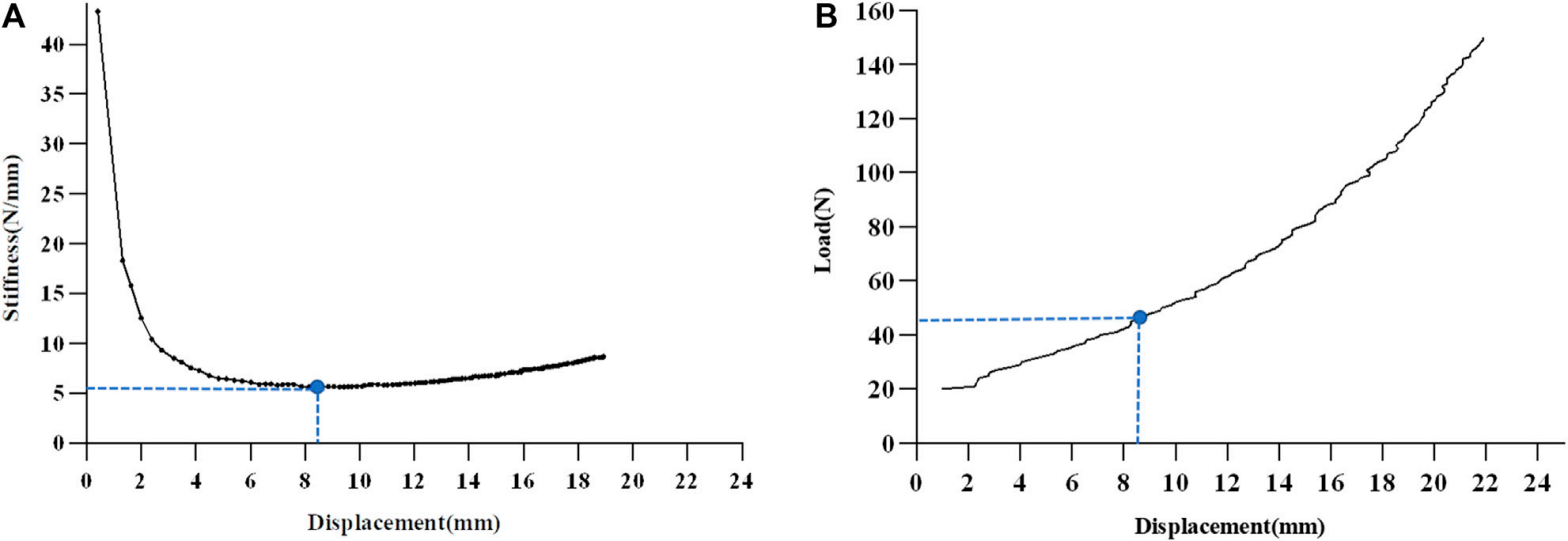
The Schematic diagram of load-displacement curve division. **(A)** The load-displacement curve is divided into two regions, using the group mean minimum value of stiffness as the turning point. **(B)** The restricting contribution of the anterior cruciate ligament transforms the displacement-stiffness curve from a sharp decrease to a stable increase, resulting in a minimum stiffness value. *ACL* anterior cruciate ligament.

Continuous loads were applied up to 150 N, and the displacement corresponding to each 10N increase load was recorded. A load-displacement curve was plotted to observe the change in stiffness (Figure 2A). The stiffness value of the ACL was minimal when it functioned as a tibial restraint, and gradually increased thereafter (Figure 2B). Taking the minimum stiffness as the turning point, the load-displacement curve was divided into two regions (Figure 2A). The stiffness of region 1 was estimated as follows: 52 N divided by the corresponding anterior tibial displacement (ATD). The stiffness in region 2 was estimated as follows: 98 N (calculated as 150 N–52 N) divided by the difference in the ATD between 150 N and 52 N.

### Statistical analysis

The Kolmogorov–Smirnov test was used to verify the normality of all variables. The Wilcoxon test was used for variables that did not conform to normality. Effect sizes were calculated. Cohen’s d effect size classification was defined as 0.2 for a small effect, 0.5 for a medium effect, and 0.8 for a large effect (1990). The independent *t*-test was used to compare the displacement and instantaneous stiffness corresponding to each 10N increase load. In addition, the minimum stiffness, corresponding load, and stiffnesses in the two regions were also compared between the control and ACL injury groups. A paired sample *t*-test was used to compare the minimum stiffness, corresponding load, and stiffnesses in the two regions on the injured and healthy sides in the ACL injury group. Appropriate variables to construct the receiver operating characteristic (ROC) curve were selected, based on the group comparison results. The area under the curve (AUC) was calculated to determine the appropriate diagnostic criteria for detecting ACL injury. The sensitivity and specificity were further calculated. Statistical analyses were conducted using SPSS software (version 23.0; IBM, Armonk, NY, United States). The significance level was set at *p* < 0.05.

## Results

The 30 patients included 13 patients had left-sided ACL injuries and 17 patients had right-sided ACL injuries, comprised 22 men and 8 women, aged 23 ± 3.3 years, with a mean body mass index of 21.1 kg/m2. The control group comprised 17 men and 13 women, aged 22 ± 3.0 years, with a mean body mass index of 20.9 kg/m2. There were no statistical differences in gender (*p* = 0.18), age (*p* = 0.19), or body mass index (*p* = 0.79) between the two groups of participants.

The mean ± standard deviation of displacements, corresponding to each 10N increase in load between the ACL injury group and the control group, are shown in Table 1. The displacement was significantly greater in the ACL injury group (all *p* < 0.001), with the largest effect size observed at the load of 150 N (effect size = 2.2).

**TABLE 1:** Comparison of ATD in the ACL injury group and the control group.

Loads (N)	ACL injury group (mm)	Control group (mm)	*p*-Value	Effect size
30	4.5 ± 1.1	3.8 ± 0.5	0.001^**^	0.8
40	7.4 ± 1.8	6.3 ± 0.7	0.001^**^	1.2
50	9.7 ± 2.1	8.2 ± 0.9	0.000^***^	1.4
60	11.1 ± 2.2	9.8 ± 1.1	0.000^***^	1.6
70	13.5 ± 2.3	11.0 ± 1.2	0.000^***^	1.7
80	14.9 ± 2.2	12.1 ± 1.3	0.000^***^	1.7
90	16.3 ± 2.3	13.0 ± 1.4	0.000^***^	1.8
100	17.7 ± 2.3	13.9 ± 1.5	0.000^***^	1.8
110	18.7 ± 2.4	14.7 ± 1.6	0.000^***^	1.9
120	19.8 ± 2.5	15.4 ± 1.7	0.000^***^	2.0
130	20.8 ± 2.4	16.0 ± 1.7	0.000^***^	2.1
140	21.8 ± 2.6	16.6 ± 1.8	0.000^***^	2.1
150	22.6 ± 2.6	17.2 ± 1.9	0.000^***^	2.2

ATD: anterior tibial displacement.

Data are reported as M ± SD; the level of significance was established *a priori* at *p* < 0.05.

**p < 0.01.

***p < 0.001.

**FIGURE 3:**
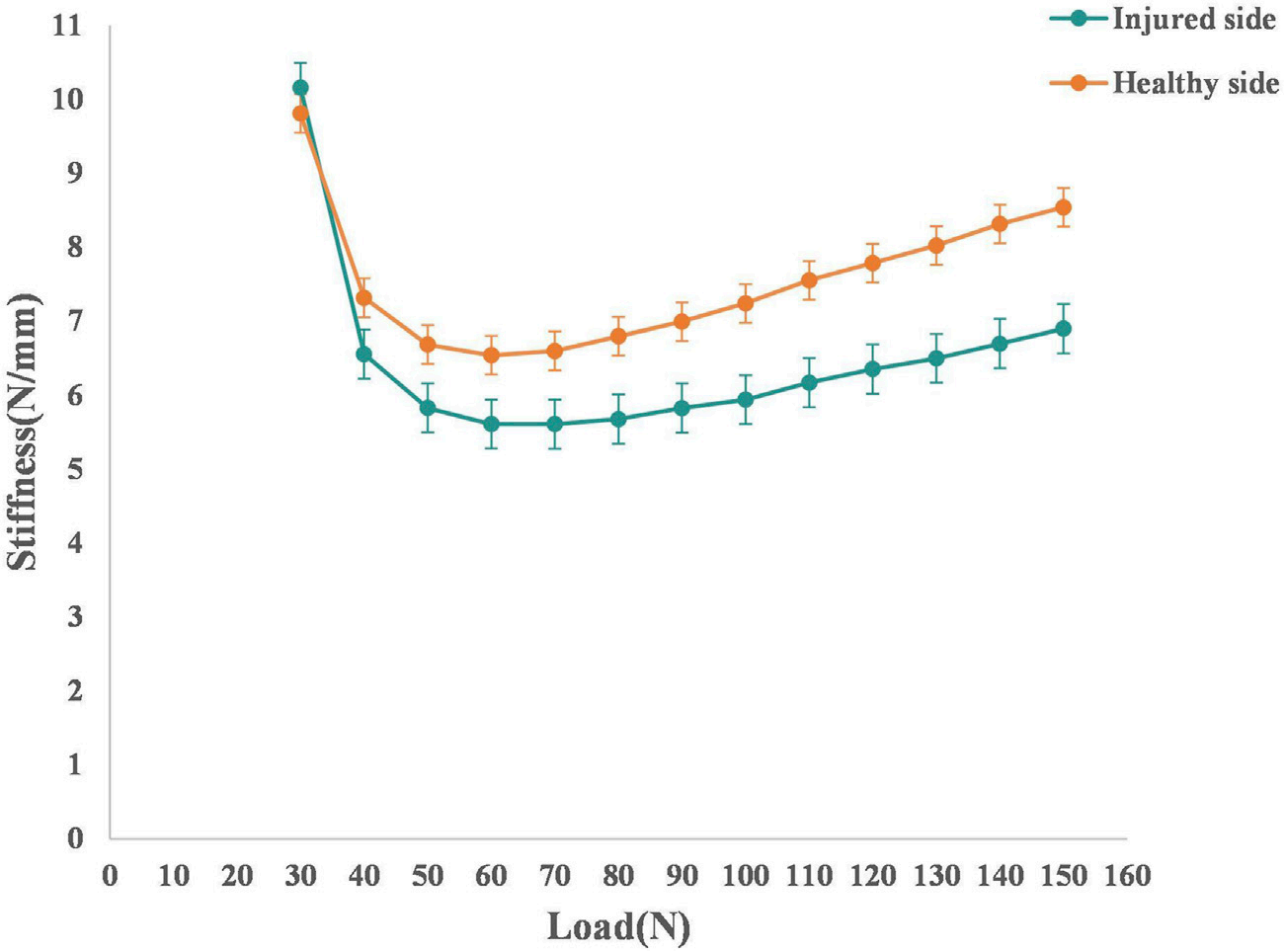
The average load-stiffness curves of the injured and healthy sides in the ACL injury group. Both groups have 30 knees *ACL* anterior cruciate ligament.

The comparison results of instantaneous stiffness on the injured and healthy sides in the ACL injury group are shown in Figure 3. The stiffness was significantly lower on the injured side (all, *p* < 0.001), with the largest effect size at the load of 150 N (effect size = 1.5).

The comparison results of the minimum stiffness, corresponding load, and stiffnesses in the two regions in the control and ACL injury groups are shown in Table 2. We observed no significant difference in the corresponding load at the minimum stiffness between the control and ACL injury groups (*p* = 0.62). The minimum stiffness of the injured side in the ACL injury group was significantly lower than that of the healthy side and the control group (all *p* < 0.05). The load-displacement curve was divided into two regions, using the minimum stiffness mean value of the two groups as the turning point. The stiffness in the two regions of the injured side in the ACL injury group was significantly lower than that of the healthy side and the control group (all *p* < 0.05). The stiffness in region 2 of the healthy side in the ACL injury group was significantly lower than that of the control group (*p* < 0.05).

**TABLE 2:** Comparison of the minimum stiffness, corresponding load, and stiffness in region 1 and region 2 between the control and ACL injury groups.

Variable	Control group	ACL injury group	
		Injured side (N/mm)	Healthy side (N/mm)
Load (N)	50.1 ± 5.2	53.0 ± 5.7	50.7 ± 6.0
Minimum stiffness (N/mm)	6.0 ± 0.7	4.9 ± 0.8*	5.8 ± 0.9**
Stiffness in region 1 (N/mm)	6.2 ± 0.7	5.4 ± 1.6*	6.2 ± 1.3**
Stiffness in region 2 (N/mm)	11.5 ± 1.6	8.1 ± 1.5*	10.4 ± 1.9*^,^ **

Data are reported as the mean ± standard deviation. The level of significance is established *a priori* at *p* < 0.05.

^*^Statistically significant difference, compared with the control group (*p* < 0.05).

^**^Statistically significant difference, compared with the injured side (*p* < 0.05).

ACL, anterior cruciate ligament.

**FIGURE 4:**
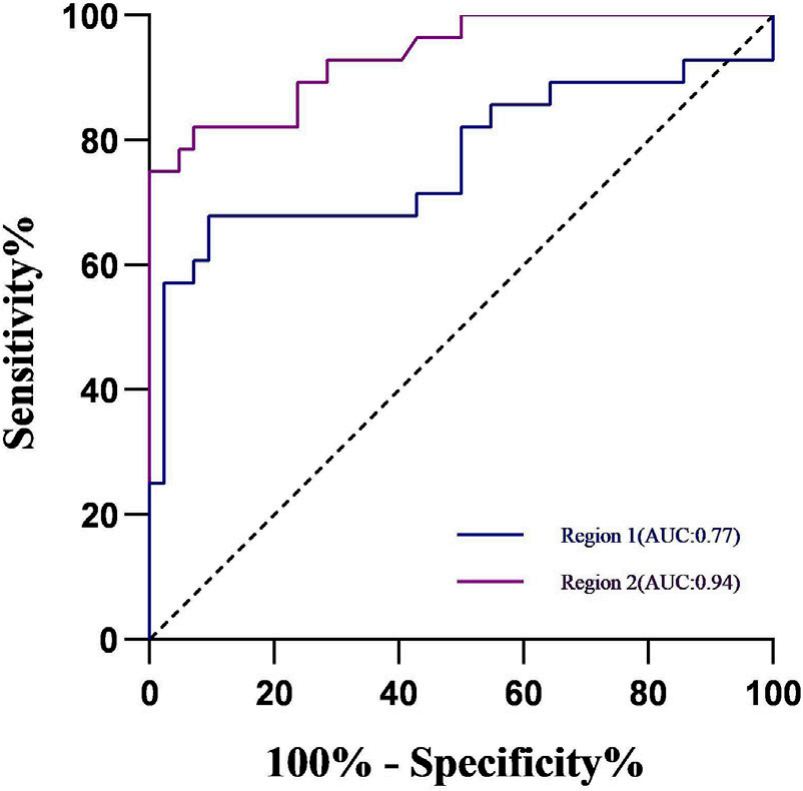
The ROC curve of stiffness in the two regions. *ROC* receiver operating curve.

ROC curves were plotted, based on changes in stiffness in the two regions in the control and ACL injury groups. The ROC curves of stiffness in the two regions are shown in Figure 4. The stiffness in region 2 had the largest AUC [0.94 (95% CI 0.88–0.99)]. The point near the upper left corner was defined as the best diagnostic criterion for detecting ACL injury, with a critical value of 9.62 N/mm. The sensitivity and specificity were 93% and 82%, respectively.

## Discussion

In this study, we used a novel digital arthrometer to measure knee laxity in patients after ACL injury. The most important finding of this study was the discovery of sensitive indicators to identify ACL injuries by conducting subregional analysis of load-displacement curves. We believe that these results will help clinicians fully understand the biomechanical properties of the ligament after injury and will provide a reference for the grading and diagnosis of ligament injuries. Our results demonstrated that stiffness in the ACL injury group was significantly lower than that of the control group in both defined regions. After ACL injury, Paterno et al. (2012) observed a 15- to 25-fold increase in the risk of contralateral ACL injury. In our study, the ACL injury group, compared to the control group, had significantly lower stiffness on the healthy side in region 2. This finding indicated an earlier onset of mechanical changes in ligament properties among patients with ACL injuries, compared to these changes in healthy individuals. We plotted ROC curves to reflect the change in stiffness under different subregions. Region 2 had the largest AUC (0.94) at a cut-off value of 9.62 N/mm, and a sensitivity and specificity of 93% and 82%, respectively. The observed changes in ACL stiffness within region 2 may reflect preinjury mechanical properties. Therefore, the findings of this study support our initial hypothesis that alterations in stiffness at various regions along the load-displacement curve could serve as a clinical indicator for identifying ACL injuries.

KT1000 and KT2000 are widely used arthrometers. A meta-analysis by van Eck et al. (2013a) showed that, the higher the applied load, the higher was the diagnostic power. In this aforementioned study, the average sensitivity increased by 39% (54%–93%) from 69N to 89N to the manual maximum load. KT1000 and KT2000 arthrometers have been used to establish widely accepted diagnostic criteria; for example, a side-to-side difference greater than 3 mm at 134 N was considered a good diagnostic value (Daniel et al., 1985b; Rangger et al., 1993). However, side-to-side differences usually use the uninjured knee as the reference, which makes accurately assessing knee laxity in patients with bilateral knee injuries difficult. In addition, in a study of knee laxity among healthy individuals, a side-to-side difference of greater than 3 mm was found in 2% of healthy individuals (Daniel et al., 1985a). Markolf et al. (1976); Markolf et al. (1984) previously suggested that stiffness could be an important indicator for assessing ligament injury. They used the KT2000 arthrometer to obtain ATD data from controls (*n* = 21) and ACL-injured patients (*n* = 6) to assess stiffness changes. Their results were that patients with ACL injuries (3.8 N/mm) had significantly lower stiffness values than did healthy individuals (23.2 N/mm) at a load of 134 N, which is in accordance with the results of our study. Instantaneous stiffness was lower in ACL patients than in healthy participants (*p* < 0.05). Therefore, the stiffness characteristics can objectively reflect the biomechanical properties of the ACL and can be used to differentiate the injured knee from the healthy knee.

In a study by Eagar et al. (2001), which used the KT2000 quantitative Lachman’s ACL injury test, the authors found that the minimum stiffness value corresponded to a mean load of 49 N at different knee flexion angles. This finding was similar to the results of our study wherein the mean load at the turning point between the two regions of the two groups was 52 N. Before the turning point, the applied load causes directional compression of the soft tissues of the lower leg to overcome the weight of the lower leg (Minns et al., 1973; Maitland et al., 1995). After the turning point, the stiffness curve tended to flatten in response to the restraining effect of the ACL on the tibia (Figure 2B). We plotted ROC curves reflecting the change in stiffness under different subregions. Region 2 had the largest AUC (0.94), with a cut-off value of 9.62 N/mm and sensitivity and specificity of 93% and 82%, respectively. Therefore, we suggest that the stiffness characteristics after the turning point (region 2) can objectively reflect the biomechanical properties of the ACL and can be used to differentiate the ACL-injured knee from the healthy knee.

In the present study, we compared the ATD 10-N interval between the injured and healthy knee in the ACL injury group. The ATD of the healthy knee was significantly lower than that of the injured knee. In the case of ACL injury, ATD exhibits a significant increase due to loss of restraint, thereby resulting in an alteration in the slope of the stiffness-displacement curve. Typical curves have an initial low stiffness linear region and an end high stiffness linear region with transitional nonlinear regions in between (Markolf et al., 1976). With increasing load, the load-displacement curve inflects because of the limiting effect of the ACL on ATD, at which point the minimum value of stiffness occurs (Figure 2A). In the present study, the mean load at the turning point between the two regions of the two groups was 52 N. In addition, the minimum stiffness of the ACL injury group was significantly lower than that of the control group. The mean displacement of the control group under the 52N load was interestingly 8.6 mm, whereas the displacement on the healthy side in the ACL injury group was 8.7 mm. However, in region 2, the stiffness of the healthy knee in the ACL group was significantly lower than that of the control group (*p* < 0.05). The decrease in stiffness indicated a change in the ACL constraint of the ACL on the tibia (Liu et al., 2002). These features can provide a reference point for the differentiation between normal and ACL-injured knees. We hypothesized that alterations in the stiffness of region 2 may serve as a significant predictor for ACL injury and offer novel insights into the clinical grading of such injuries.

When it comes to clinical utility, the Ligs device offers several benefits. First of all, for the user, Ligs device is simple and convenient to operate. The straightforward and adaptable operation minimizes measurement inaccuracies that the examiner may have caused. Previous studies have confirmed the excellent intra- and inter-rater reliability (intraclass correlation, >0.9) of the Ligs arthrometer (Chen et al., 2022; Wu et al., 2023). Second, for the patient, the operation is more flexible because the rocker at the end of the main unit controls the load application. The loading can be customized for each participant during the test based on their acceptable range. Above all, Ligs comes equipped with integrated load and displacement sensors that have a 30Hz sampling frequency. As a result, frame by frame recording of real-time changes in load and displacement is possible. Furthermore, there are no scenario limitations when using the device due to its portable size and the short time required for testing.

This study has limitations which should be considered. First of all, in our study, the participants were patients who were prepared to undergo ACL reconstruction surgery, so the mechanical properties of each part of the structure were not studied in depth enough and were limited to the macroscopic stiffness changes of the knee joint. However, the knee joint has a complex structural composition, including tissues with different biomechanical properties such as the joint capsule, ligaments, and menisci. The tensile energy dissipation and mechanical properties of the meniscus are highly dependent on the direction of loading, as demonstrated in the study of the mechanical properties of the meniscus by Morejon et al. (2023). In addition, the water content of the meniscus is inversely related to its energy dissipation. Accordingly, the effects of ACL injury on the mechanical properties of different knee joint structures should be analyzed in depth in future studies. Secondly, in our study, only patients with isolated ACL injuries were included. For patients with combined meniscal injuries of different types and levels, the loading settings of the Ligs device and the effect on outcomes are not clear. Zhang et al. (2016) confirmed in a study that the location of the meniscal rupture modifies knee kinematics in patients with ACL combined with meniscal injuries. Musahl et al. (2016) found that a concomitant injury to the anterolateral capsule, medial meniscus, or lateral meniscus is associated with increased knee rotatory laxity in patients with an ACL injury. In a study by Massey et al. (2019) on the failure loads of different suture repair techniques after meniscal tears, the results showed that the failure loads of the parallel technique, cross-stitch technique and rebar repair of radial meniscal tears were 85.5 N ± 22.0, 76.2 N ± 28.8, and 124.1 N ± 27.1, respectively. Therefore, individuals should be more accurately categorized according to the type of injury in future studies to further investigate the mechanisms by which meniscal injuries alter joint biomechanics. In addition, future studies should evaluate patients undergoing different reconstructive procedures. And then, we only included patients with a complete ACL injury. Patients with different ACL injury classifications were therefore not studied in detail. Further research is needed to investigate whether load-displacement curves can be used to classify ACL injuries. In addition, our study was limited to healthy college students and adults. The results of the study are not applicable to adolescent and child patients. Future studies should fully consider the ligament laxity characteristics of different age groups to expand the applicability of the findings.

## Conclusion

In the present study, we used a novel digital arthrometer to investigate the ligament stiffness characteristics after ACL injury. This study demonstrated that the two regions of the load-displacement curve following ACL injury have significantly different biomechanical characteristics. These results provide new insights into the laxity characteristics of the knee after ACL injury. Based on the results of the present study, we propose that a stiffness value less than 9.62 N/mm is a new diagnostic reference. We believe that this indicator may be a useful for identifying knee laxity in the later stages of increased loading.

## Data Availability

The raw data supporting the conclusions of this article will be made available by the authors, without undue reservation.
